# LONGITUDINAL ASSOCIATIONS BETWEEN HAND GRIP STRENGTH AND DEPRESSIVE SYMPTOMS AMONG OLDER ADULTS USING KLOSA

**DOI:** 10.1093/geroni/igae098.4016

**Published:** 2024-12-31

**Authors:** Sooyoung Kwon, Gwang Suk Kim

**Affiliations:** YONSEI UNIVERSITY, Seoul, Republic of Korea; Yonsei University, Seoul, Republic of Korea

## Abstract

Many studies have reported an inverse association of hand grip strength (HGS) and depressive symptoms. However, the longitudinal directionality between these two factors remains unclear. This study was performed using a random intercept cross-lagged panel model to investigate the longitudinal reciprocal associations and autoregressive relationships between HGS and depressive symptoms in older Korean adults. We conducted secondary data analysis using the data of 3,353 older adults (mean age=74.38±6.484 [range:65~101] years; 1,480 [44.1%] were men at baseline) from the Korean Longitudinal Study of Aging (KLoSA) across 2016, 2018, 2020, and 2022 (four waves). Measures included general characteristics, mean values of HGS, and the Center for Epidemiologic Studies Depression scale-10. Result showed that no significant cross-lagged path was observed between HGS and depressive symptoms, however, across individuals, HGS was inversely correlated with depressive symptoms (β=-9.041, p<.001), and the concurrent negative relationship between these two conditions was significant only at baseline (β=-1.578, p=.004), indicating that individuals with lower HGS had higher depressive symptoms. All autoregressive paths were significant except from 2020 to 2022, suggesting the severities in one’s level of HGS and depressive symptoms carried over to the next time point. When considering gender differences, the same pattern of HGS and depressive symptoms was observed in men, while women showed a partially inverse association between them. Our findings suggest that HGS and depressive symptoms have a negative cross-sectional relationship, and gender has a different effect on the longitudinal correlation between HGS and depressive symptoms.

